# Ethanol Extract from* Ulva prolifera* Prevents High-Fat Diet-Induced Insulin Resistance, Oxidative Stress, and Inflammation Response in Mice

**DOI:** 10.1155/2018/1374565

**Published:** 2018-01-03

**Authors:** Wei Song, Zongling Wang, Xuelei Zhang, Yan Li

**Affiliations:** ^1^Key Laboratory of Science and Engineering for Marine Ecology and Environment, The First Institute of Oceanography, SOA, Qingdao 266061, China; ^2^Laboratory of Marine Ecology and Environmental Science, Qingdao National Laboratory for Marine Science and Technology, Qingdao 266235, China

## Abstract

*Ulva prolifera* is the major causative species in the green tide, a serious marine ecological disaster, which bloomed in the Yellow Sea and the Bohai Sea of China. However, it is also a popular edible seaweed and its extracts exerts anti-inflammatory and antioxidant effects. The present study investigated the effects of ethanol extract of* U. prolifera *(EUP) on insulin sensitivity, inflammatory response, and oxidative stress in high-fat-diet- (HFD-) treated mice. HFD-treated mice obtained drinking water containing 2% or 5% EUP. The results showed that EUP supplementation significantly prevented HFD-induced weight gain of liver and fat. EUP supplementation also improved glucose tolerance and insulin resistance in HFD-treated mice. Moreover, EUP supplementation prevented the increased expression of genes involved in triglyceride synthesis and proinflammatory genes and the decreased expression of genes involved in fatty acid oxidation in liver of HFD-treated mice. Furthermore, EUP supplementation decreased reactive oxygen species content, while increasing glutathione content and glutathione peroxidase activity in HFD-treated mice. In conclusion, our results showed that EUP improved insulin resistance and had antilipid accumulation and anti-inflammatory and antioxidative effects on HFD-treated mice. We suggested that* U. prolifera *extracts may be regarded as potential candidate for the prevention of nonalcoholic fatty liver disease.

## 1. Introduction

*Ulva prolifera*, a green macroalgae species which recurrently bloomed in the Yellow Sea of China, causes the world's largest green tides from 2008 [1]. Since 2015, new green tides events begin to bloom near the Beidaihe Scenic in the Bohai Sea, and* U. prolifera *is also the major causative species. These ecological disasters have caused serious influences in tourism, aquaculture, and marine ecosystems [2, 3]. Alternative uses of biomass to profit from the green tide events are effective methods to offset the bill for environmental damage.* U. prolifera* is mainly used as food or for medical purposes, because it is rich in polysaccharides, proteins, and essential mineral elements for human health, and it also has low content of fats and cellulose [4].

Consequently, increasing attention in recent years has been paid to marine macroalgae to develop new functional food ingredients for the treatment of overnutrition-induced metabolic syndrome. Abundant researches studied the antioxidant ability of the extracts from different kinds of macroalgae, such as extract from* Sargassum pallidum *[5],* Gracilaria edulis *[6], and* Ulva prolifera *[7]. However, few studies have investigated their effects on the prevention of metabolic syndrome.

Nonalcoholic fatty liver disease (NAFLD), which is the most popular liver disorder, is considered as the liver manifestation of metabolic syndrome. Overnutrition results in an imbalance of free fatty acid uptake from circulation, fatty acid de nonovo synthesis and oxidation, and triglyceride (TG) export, which further lead to hepatic TG overaccumulation [8]. The lipid metabolism disorder in liver often happened in accordance with insulin resistance, inflammatory response, and oxidative stress. NAFLD has a high prevalence in conjunction with type 2 diabetes and obesity. In this study, the marine green macroalgae,* U. prolifera*, was selected as the main experimental material to investigate its effects on insulin resistance, oxidative stress, and inflammation response in high-fat-diet-induced mice.

## 2. Materials and Methods

### 2.1. Materials and Sample Extraction

Green macroalgae,* U. prolifera*, was collected in April 2017 from the coast of Beidaihe in the Bohai Sea during the green tides development process and supplied by the Laboratory of Marine Ecology and Environmental Science, Qingdao National Laboratory for Marine Science and Technology (Qingdao, RP China). Ethanol extract from* U. prolifera* was obtained according to previous method [7]. Briefly, the raw sample was rinsed with running water, dried at 60°C, and then milled with a bender. After sieving (<0.5 mm), the sample (100 g) was extracted three times with 95% ethanol (1 L) at 60°C for 2 h. Finally, the supernatant was collected by centrifuging the solution at 18,500 g for 15 min at room temperature. The crude extract was obtained by further concentrating the supernatant using a vacuum evaporator.

### 2.2. Animal Care and Experimental Design

Thirty-two male C57BL/6J mice (9 weeks old) were housed at 22 ± 2°C with a relative humidity of 50 ± 5%, and all the animals were free to obtain food and water. All mice were randomly assigned into four groups (*n* = 8): (i) mice were fed on a low-fat diet (control); (ii) mice were fed on a high-fat diet; (iii) mice were fed on a high-fat diet (HF) supplemented with 2% (vol/vol) ethanol extract from* U. prolifera* (LUP); and (iv) mice were fed on a high-fat diet supplemented with 5% ethanol extract from* U. prolifera* (HUP). Ethanol extract from* U. prolifera* was supplemented in the drinking water. The duration of experiment was 8 weeks. The low-fat diet consisted of 10% (kcal%) fat while the high-fat diet consisted of 60% fat (Research Diets, Inc., New Brunswick, NJ, USA). At the end of the experiment, blood was taken from the retroorbital sinus after overnight fasting. Then, following cervical dislocation, liver, major subcutaneous white adipose tissue (WAT) (inguinal WAT), and two representative visceral WATs (mesenteric WAT and epididymal WAT) were collected and weighed. Liver samples were immediately frozen in liquid nitrogen and stored at −80°C. This study was approved by the animal welfare committee of The First Institute of Oceanography of China.

### 2.3. Insulin and Triglyceride Assay

Serum insulin was determined using ELISA Kit (Cusabio Biotech Co., Ltd., Wuhan, China) and triglyceride (TG) content was determined using the corresponding commercial colorimetric assay kits (Beijing Strong Biotechnologies, Inc., Beijing, China) according to the manufacturer's instructions.

### 2.4. Intraperitoneal Glucose and Insulin Tolerance Test

Intraperitoneal glucose test and insulin tolerance test were conducted 1 week before the end of the experiment. After fasting for 6 h, mice were intraperitoneally injected with a dose of 1.0 g glucose or 0.65 U insulin per kg body weight. Glucose concentration was measured at 0, 30, 60, and 120 min using a OneTouch UltraEasy glucometer.

### 2.5. Inflammatory Cytokines Determination

The concentrations of interleukin-1*β* (IL-1*β*), IL-6, and tumor necrosis factor-*α* (TNF-*α*) in serum were determined using microplate reader with ELISA kits (Wuhan Huamei Biotech Co., Ltd., Wuhan, China) according to the manufacturer's instructions.

### 2.6. Glutathione and Glutathione Peroxidase Determination

Reduced glutathione (GSH) content and glutathione peroxidase (GSH-Px) activity were determined using the colorimetric assay kits (Cayman Chemical Company, Ann Arbor, Michigan, USA) in accordance with the manufacturer's instructions.

### 2.7. Reactive Oxygen Species Determination

Reactive oxygen species (ROS) content in liver was determined according to previous study [9, 10]. Briefly, liver samples were embedded in an optimum cutting temperature compound (Sakura, Tokyo, Japan), frozen in a methylbutane-chilled bath at −80°C, and then stored in liquid nitrogen. Sections (10 *μ*m) were sliced and stained with dihydroethidium (Sigma-Aldrich, Shanghai, China) for 20 min at 37°C. Representative pictures were taken by fluorescence microscopy and pictures were analyzed by Image Browser software (Leica, Wetzlar, Germany).

### 2.8. RT-qPCR Analysis

Total RNA was isolated using the TRIzol reagent and cDNA was obtained using reverse transcriptase. RT-qPCR was performed with a total volume of 10 *μ*L solution containing 5 *μ*L SYBR Green mix, 0.2 *μ*L Rox, 3 *μ*L DEPC-treated H_2_O, 1 *μ*L cDNA template, and 0.4 *μ*L of each of the forward and reverse primers [11]. All samples were run in triplicate and the average values were calculated. Primers were showed in Table 1.

### 2.9. Statistical Analysis

All data were presented as Least Squares Means ± SEM. Data were analyzed by one-way ANOVA using the general linear model procedures and a mixed procedure (PROC MIXED) of SAS software version 9.2 (SAS Institute Inc., Cary, NC, USA). Mean values were considered to be significantly different when *p* < 0.05.

## 3. Results

### 3.1. Effects of Ethanol Extract from* U. prolifera *on Body Weight, Liver, and Fat Weight in HFD-Treated Mice

**Figure 1:**
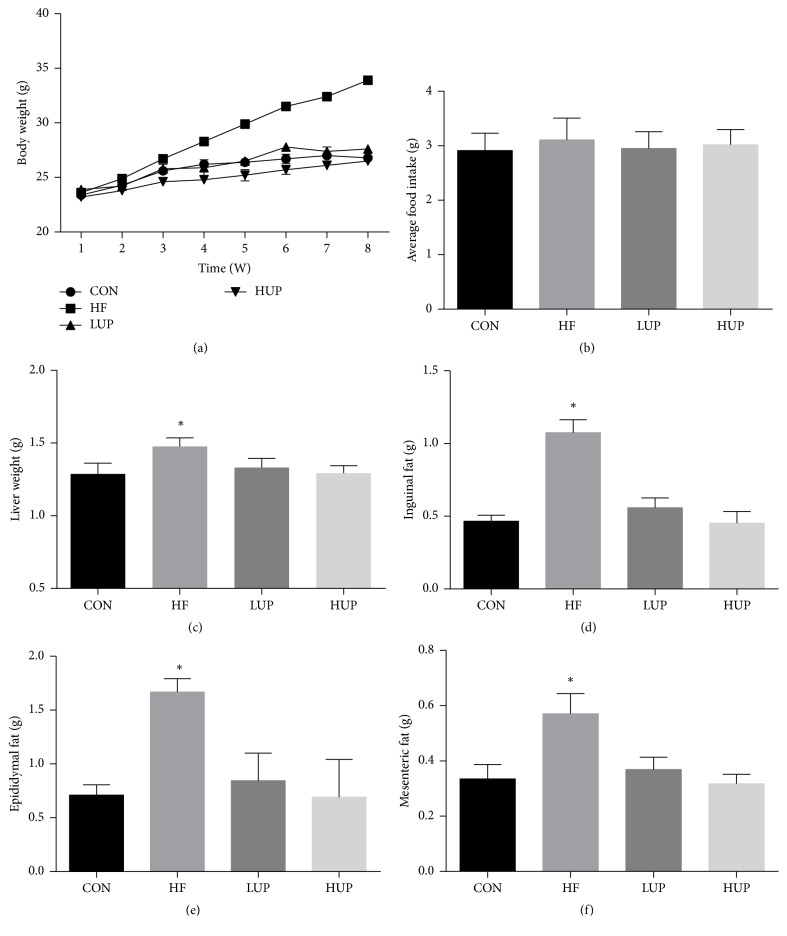
Ethanol extract from* U. prolifera *prevented body weight gain in HFD-treated mice. CON, mice were fed on a low-fat diet; HF, mice were fed on a high-fat diet; LUP, mice were fed on a high-fat diet supplemented with 2% ethanol extract from* U. prolifera*; HUP, mice were fed on a high-fat diet supplemented with 5% ethanol extract from* U. prolifera*. Values are expressed as mean ± SEM, *n* = 8; ^*∗*^*p* < 0.05; *∗* means significant difference between HF group and the other three groups.

As shown in Figure 1, body weight, liver, and fat (inguinal, epididymal, and mesenteric fat) weight were significantly higher in HFD-treated mice when compared with control mice, while there was no significant difference between mice in LUP and HUP groups and control group.

### 3.2. Effects of Ethanol Extract from* U. prolifera *on HFD-Induced Insulin Resistance

**Figure 2:**
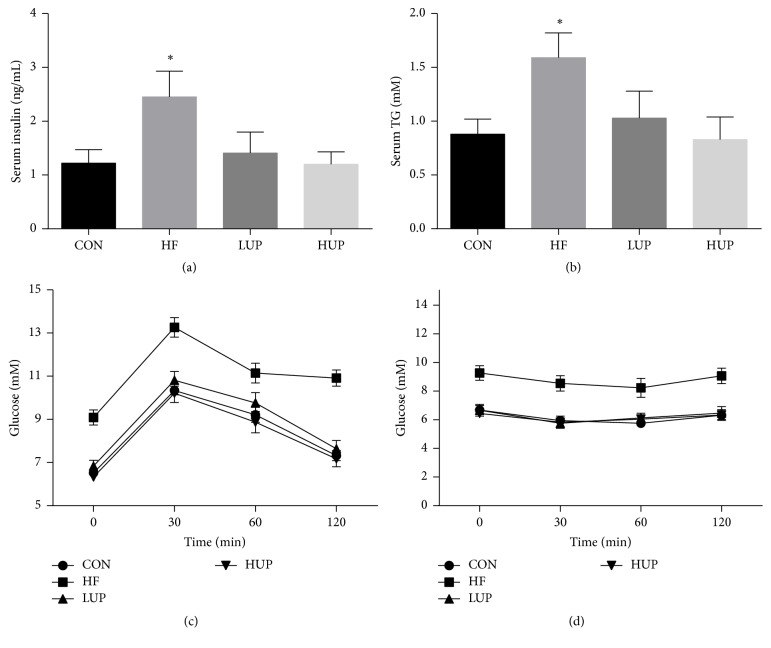
Ethanol extract from* U. prolifera *improved glucose tolerance and insulin sensitivity in HFD-treated mice. (a) Serum insulin concentration. (b) Serum TG concentration. (c) Glucose tolerance test. (d) Insulin tolerance test. CON, mice were fed on a low-fat diet; HF, mice were fed on a high-fat diet; LUP, mice were fed on a high-fat diet supplemented with 2% ethanol extract from* U. prolifera*; HUP, mice were fed on a high-fat diet supplemented with 5% ethanol extract from* U. prolifera*. TG, triglyceride. Values are expressed as mean ± SEM, *n* = 8; ^*∗*^*p* < 0.05; *∗* means significant difference between HF group and the other three groups.

As shown in Figure 2, serum insulin and TG contents were significantly higher in HFD-treated mice when compared with control mice, while there was no significant difference between mice in LUP and HUP groups and control group. HFD-treated mice showed remarkably impaired glucose tolerance and insulin sensitivity, while supplementation with ethanol extract from* U. prolifera *prevented these changes induced by HFD.

### 3.3. Effects of Ethanol Extract from* U. prolifera *on HFD-Induced Inflammatory Response

**Figure 3:**
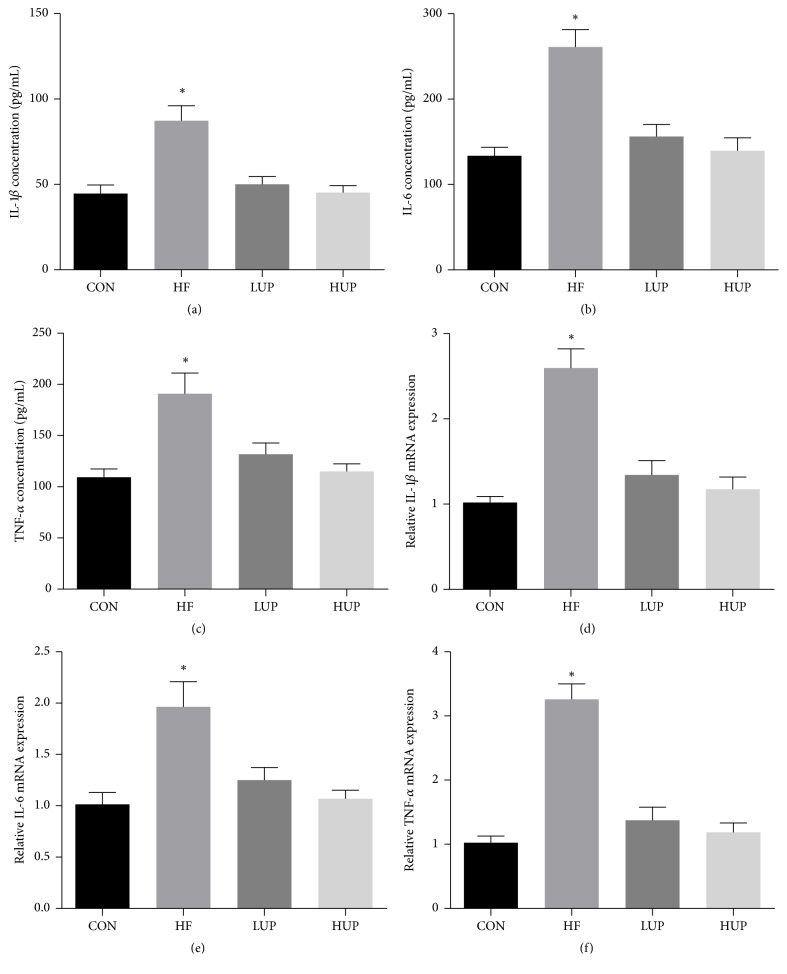
Ethanol extract from* U. prolifera *prevented inflammatory response in liver of HFD-treated mice. CON, mice were fed on a low-fat diet; HF, mice were fed on a high-fat diet; LUP, mice were fed on a high-fat diet supplemented with 2% ethanol extract from* U. prolifera*; HUP, mice were fed on a high-fat diet supplemented with 5% ethanol extract from* U. prolifera*. Values are expressed as mean ± SEM, *n* = 8; ^*∗*^*p* < 0.05; *∗* means significant difference between HF group and the other three groups.

As shown in Figure 3, serum IL-1*β*, IL-6 and TNF-*α* concentrations, as well as gene expression of IL-1*β*, IL-6 and TNF-*α* in liver, were significantly higher in HFD-treated mice when compared with control mice, while there was no significant difference between mice in LUP and HUP groups, and control group.

### 3.4. Effects of Ethanol Extract from* U. prolifera *on HFD-Induced Oxidative Stress

**Figure 4:**
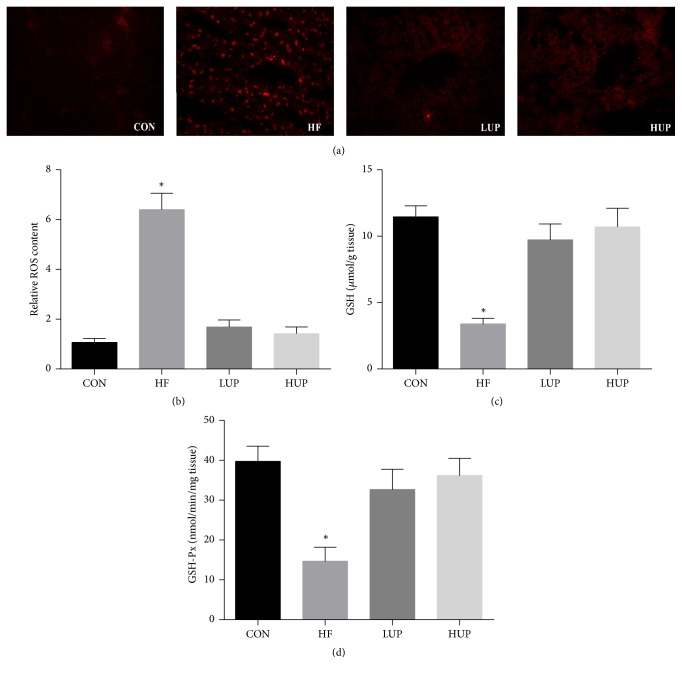
Ethanol extract from* U. prolifera *improved oxidative stress in liver of HFD-treated mice. (a) ROS stained with dihydroethidium in liver (red). (b) Relative ROS content; (c) GSH content in liver; (d) GSH-Px activity in liver. CON, mice were fed on a low-fat diet; HF, mice were fed on a high-fat diet; LUP, mice were fed on a high-fat diet supplemented with 2% ethanol extract from* U. prolifera*; HUP, mice were fed on a high-fat diet supplemented with 5% ethanol extract from* U. prolifera*. ROS, reactive oxygen species; GSH, glutathione; GSH-Px, glutathione peroxidase. Values are expressed as mean ± SEM, *n* = 8; ^*∗*^*p* < 0.05; *∗* means significant difference between HF group and the other three groups.

As shown in Figure 4, ROS content in liver was significantly higher, while GSH content and GSH-Px activity were significantly lower in HFD-treated mice when compared with control mice. However, there was no significant difference between mice in LUP and HUP groups, and control group.

### 3.5. Effects of Ethanol Extract from* U. prolifera *on HFD-Induced Lipid Accumulation

**Figure 5:**
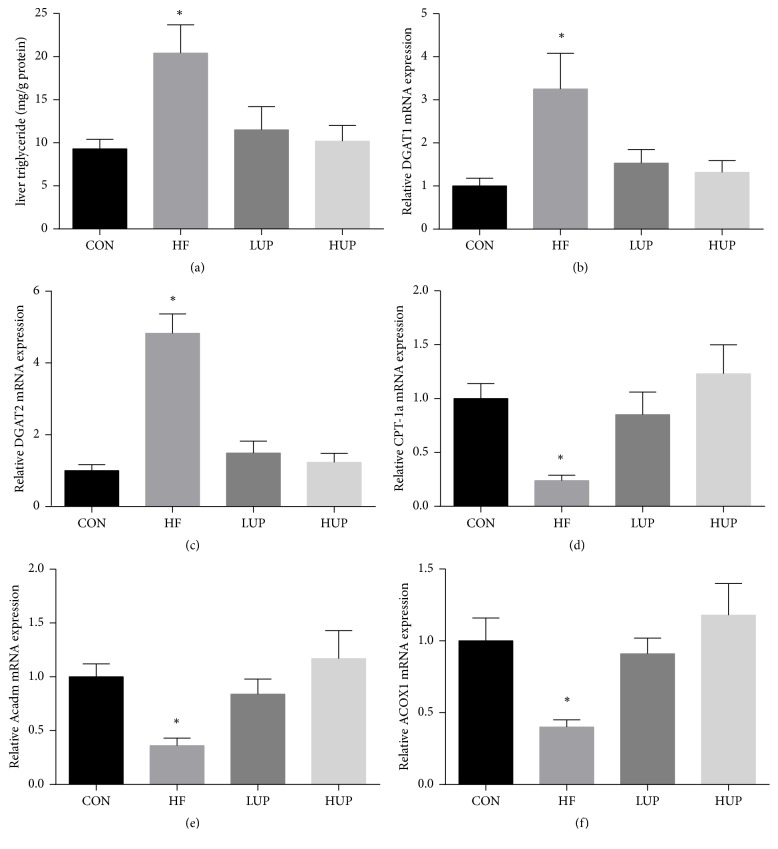
Ethanol extract from* U. prolifera *prevented lipid accumulation in liver of HFD-treated mice. CON, mice were fed on a low-fat diet; HF, mice were fed on a high-fat diet; LUP, mice were fed on a high-fat diet supplemented with 2% ethanol extract from* U. prolifera*; HUP, mice were fed on a high-fat diet supplemented with 5% ethanol extract from* U. prolifera*. DGAT, diacylglycerol O-acyltransferase; CPT-1a, carnitine palmitoyltransferase 1a; Acadm, medium-chain acyl-CoA dehydrogenase; ACOX1, acyl-CoA oxidase 1. Values are expressed as mean ± SEM, *n* = 8; ^*∗*^*p* < 0.05; *∗* means significant difference between HF group and the other three groups.

As shown in Figure 5, hepatic TG content, and gene expression of diacylglycerol O-acyltransferase 1 (DGAT1) and DGAT2 were significantly higher in HFD-treated mice when compared with control mice, while there was no significant difference between mice in LUP and HUP groups and control group. Gene expressions of carnitine palmitoyltransferase 1a (CPT-1a), medium-chain acyl-CoA dehydrogenase (Acadm), and acyl-CoA oxidase 1 (ACOX1) were significantly lower in HFD-treated mice when compared with control mice, while there was no significant difference between mice in LUP and HUP groups and control group.

## 4. Discussion

*U. prolifera *is rich in protein, essential amino acids, and trace elements, which make it an edible food for the coastal people from a long time ago. Recent studies further analyzed its nutritional composition and found that* U. prolifera *is rich in polysaccharide, polypeptide, alkaloids, and polyphenols [12]. In addition,* U. prolifera *has a high percent of polyunsaturated fatty acids [13]. These results indicated that* U. prolifera *might have beneficial effects on metabolic syndromes and consequently we conducted the present study to explore the effects of ethanol extract from* U. prolifera* on HFD-treated mice. The results showed that ethanol extract from* U. prolifera *could prevent the occurrence of inflammatory response, oxidative stress, and lipid accumulation in liver.

According to the “two-hit theory,” the first hit of the pathogenesis of NAFLD is represented by an accumulation of ectopic fat [14]. This lipotoxicity is considered as the driving force of NAFLD progression. As a result, we first detected body weight and liver and fat weight of HFD-treated mice. The results showed that ethanol extract from* U. prolifera *prevented these weight increases induced by HFD. And mice treated with drinking water contains either 2% or 5% ethanol extract from* U. prolifera *had the same effects. Meanwhile, we also found that TG content in both serum and liver did not increase in HFD-treated mice supplemented with ethanol extract from* U. prolifera*. These results were similar to previous study in which the hypolipidemic activity of the polysaccharides extracted from* U. prolifera* was demonstrated [15]. To further explore how ethanol extract from* U. prolifera *decreased fat accumulation, we analyzed expression of genes involved in lipid metabolism. The results showed that ethanol extract from* U. prolifera* alleviated the increases of genes involved in TG synthesis (DGAT1 and DGAT2) and decreases of genes involved in fatty acid oxidation (CPT-1a, ACOX1, and Acadm) in HFD-treated mice. These results suggested that ethanol extract from* U. prolifera *prevented ectopic lipid accumulation and it may exert this effect primarily by improving hepatic fatty acid oxidation. In addition, insulin resistance is also an early finding in metabolic syndrome and the primary pathogenic factor in the development of NAFLD. We found that ethanol extract from* U. prolifera *improved HFD-induced glucose tolerance and insulin resistance. According to previous study, polysaccharides from* U. prolifera *may play a critical role in the improvement of glucose metabolism [16].

The second hit is represented by chronic symptoms, including enhanced generation of reactive oxygen species (ROS) and increased secretion of proinflammatory responses [17]. The* in vitro* antioxidant activity of extracted product from* U. prolifera* has been proved previously by determining its radical scavenging capabilities [7, 18]. Moreover, polysaccharides from* U. prolifera *could decrease malondialdehyde content and increase superoxide dismutase and glutathione peroxidase activity [16]. Based on these results, we further examined ethanol extract from* U. prolifera *on hepatic oxidative stress in HFD-treated mice and the results showed that it decreased ROS content and increased glutathione content and glutathione peroxidase activity [19]. The potential anti-inflammatory effects of extract from* U. prolifera *have also been demonstrated both* in vitro* and* in vivo *[20, 21], and pheophytin is supposed to play the critical effects [21]. Our results further proved the anti-inflammatory ability of ethanol extract from* U. prolifera *as it prevented HFD-induced inflammation in liver.

In conclusion, our results showed that ethanol extract from* U. prolifera *could improve glucose tolerance and insulin sensitivity and had the antilipid accumulation, anti-inflammatory, and antioxidative effects on HFD-treated mice. We suggested that* U. prolifera *extracts may be regarded as potential candidates for the prevention of NAFLD. These beneficial effects would underpin the commercial exploitation of the biomass and decrease environmental damage by* U. prolifera *blooming.

## Figures and Tables

**Table 1:** 

Gene name	Primer sequences (5′-3′)
18s rRNA	F: TAACCCGTTGAACCCCATT
	R: CCATCCAATCGGTAGTAGCG
IL-1*β*	F: TGCCACCTTTTGACAGTGATG
	R: AAGGTCCACGGGAAAGACAC
IL-6	F: CCTCTCTGCAAGAGACTTCCAT
	R: AGTCTCCTCTCCGGACTTGT
TNF-*α*	F: ATGAGAAGTTCCCAAATGGC
	R: CTCCACTTGGTGGTTTGCTA
DGAT1	F: TTCCGCCTCTGGGCATT
	R: AGAATCGGCCCACAATCCA
DGAT2	F: AGTGGCAATGCTATCATCATCGT
	R: TCTTCTGGACCCATCGGCCCCAGGA
CPT-1a	F: CAGTCGACTCACCTTTCCTG
	R: CATCATGGCTTGTCTCAAGTG
Acadm	F: CTCTCTATGGGATCAGCCAGAA
	R: CCACTCAAACAAGTTTTCATACACA
ACOX1	F: TGCTCGCAGAAATGGCGATGA
	R: CAATGTGCTCACGAGCTATGA
